# Changes of Femoral Photolethysmographic Waveform Characteristics in Anesthetized Dogs with Increased Blood Pressure Induced by Epinephrine

**DOI:** 10.3389/fphys.2016.00404

**Published:** 2016-09-14

**Authors:** Hong Tang, Chengyu Liu

**Affiliations:** ^1^Department of Biomedical Engineering, Dalian University of TechnologyDalian, China; ^2^Institute of Biomedical Engineering, School of Control Science and Engineering, Shandong UniversityJinan, China

**Keywords:** pulse waveform morphology, blood pressure, Gaussian fitting model, femoral artery pulse, correlation analysis

## Abstract

**Background:** Blood pressure (BP) has been proven to play an important role in changes of the morphology of a pulse waveform. However, the extent of change of the morphology because of BP signaling has yet to be accurately confirmed.

**Objectives:** This study aims to disclose the accurate effect of BP on the changes in the morphology of the pulse waveform.

**Methods:** Two dogs' invasive intraventricular BP (varied by ejecting different doses of epinephrine) and their femoral arterial pulse waveform (FAPW) signals were synchronously recorded. For each BP increase, a normalized single cardiac beat pulse from the FAPW signal was fitted by five Gaussian curves and the changes in the Gaussian parameters (height, peak position, and time support) were observed.

**Results:** The height parameter increased while the position and time support parameters decreased with increasing systolic BP (SBP). The height ratio and the peak intervals between the first two components decreased with increasing SBP.

**Conclusions:** These results may contribute to the better understanding of the underlying changes of arterial pulse properties at different BP levels and demonstrate the potential application value of the Gaussian fitting method for clinically assessing pulse morphology and evaluating the well-being of artery system.

## Introduction

**Figure 1:**
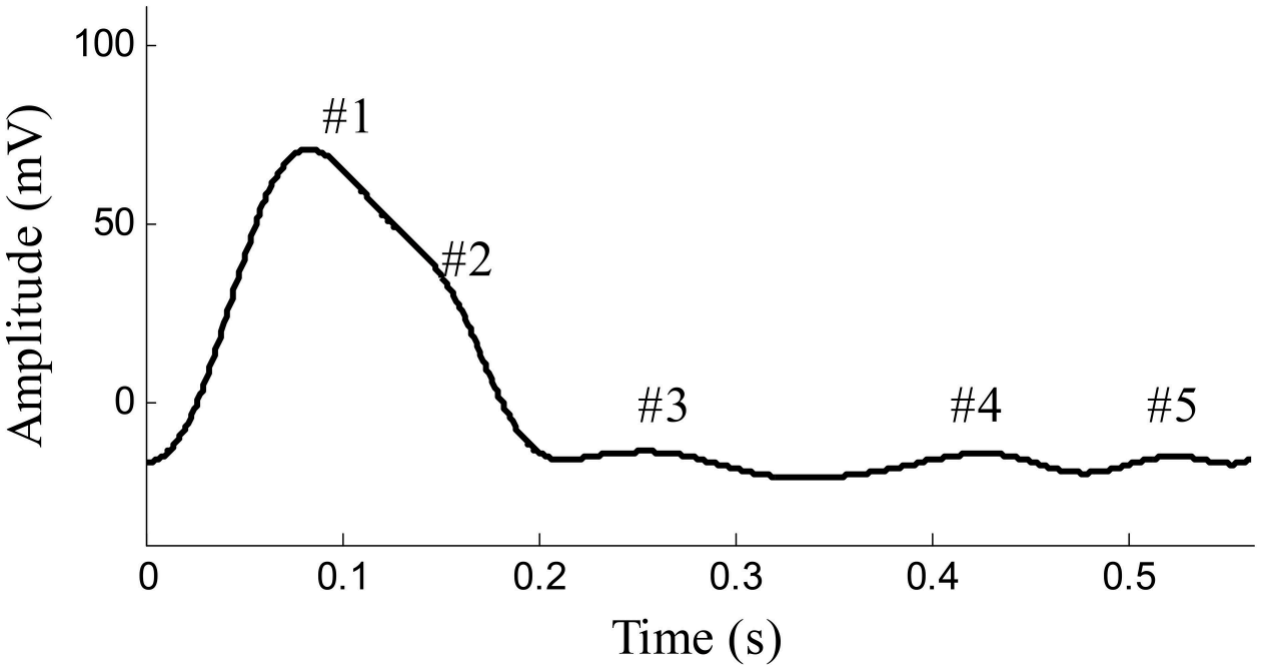
**Example of pulse waveform in one cardiaccycle recorded from the femoral artery of a beagle**. #1 Shows the forward component and #2–#5 show the reflection components of pulse waveform.

Arterial pulses are produced by the effects of cardiac ejection and the mechanical properties of the systemic arteries. Pulses first propagate through the arterial tree from the heart to the distal arteries; they are then reflected from proximal parts. Previous studies have confirmed that a surface-recorded pulse waveform is a superposition of both forward and backward components (Latham et al., 1985; Ting et al., 1990; Baruch et al., 2011). As shown in Figure 1, the forward component is derived from the blood ejection of the left ventricle, while the reflection components originate from the different reflection sites in the arterial system. The reflection sites are usually marked by significant decreases in diameter and changes in elasticity. Arterial pulse waveforms are believed to contain important physiological and pathological information on the cardiovascular system, such as information about the mechanical properties and arterial stiffness, both of which are used by clinicians for assessing the well-being of the cardiovascular system, and for the early prediction of cardiovascular diseases (London et al., 2001; Mitchell et al., 2004; Weber et al., 2004, 2005; Chirinos et al., 2005; O'Rourke and Nichols, 2005; Baruch et al., 2011; Liu et al., 2013). Existing studies have shown an increased reflection component of an arterial pulse to be an independent predictor for cardiovascular mortality (London et al., 2001), and that an increased reflection component is linked to premature coronary artery disease (Weber et al., 2004, 2005; Chirinos et al., 2005). Thus, accurately quantifying the morphology of the arterial pulse has great clinical significance.

Mitchell et al. reported an age-related increase in aortic stiffness and forward wave amplitude (Mitchell et al., 2004). O'Rourke and Nichols (2005) reported both age- and blood pressure-related increases in wave reflection. Baruch et al. (2011) found that the interval between the forward and backward components was significantly correlated with pulse pressure. The position of the main reflection component occurs early with the increase of systolic blood pressure (SBP) (Liu et al., 2014a). Blood pressure (BP) has been proven to have an important role in changing the forward and backward components of pulse waveform. However, previous studies that aimed to study the effect of BP on changes to pulse components, usually obtained BP values by using a non-invasive BP monitoring method of cuff inflation and deflation; the BP measurements usually occurred before or after the pulse waveform recording. These existing studies have lacked invasive intraventricular BP recording during pulse waveform collection. Hence, in the present study, we synchronously recorded the invasive intraventricular BP signal via a catheter-coupled pressure transducer located at the left ventricle and a peripheral artery pulse signal, and aimed to disclose the accurate effect of BP on changes to the morphology of a pulse waveform. In differing from previous studies that obtained a large BP range by using different individuals or groups, we obtained a relative large BP range from beagles by ejecting different doses of epinephrine to induce varied BP values. Furthermore, the BP values in this study were measured in the left ventricle, which differs from the BP values in previous studies commonly measured in a peripheral artery. The origin of pulse wave is in the contraction of the left ventricle. Thus, to obtain the accurate BP values, we used an invasive BP measurement method, i.e., directly inserting a catheter into the left ventricle of the anesthetized dogs.

In recent years, the Gaussian fitting method has been used for the mathematical realization of decomposing the pulse waveform into components (Liu et al., 2013, 2014a,b,c; Wang et al., 2013). The pulse waveforms were fitted by multi-Gaussian curves and the parameters (temporal location, height, and time support width) of the Gaussian curves carried information on the forward and backward components. Traditional Gaussian fitting methods usually employ three Gaussian functions to fit the pulse waveforms collected from human carotid and radial arteries with good fitting accuracy. However, in this study, during the recording of pulse waveforms from the beagles' femoral arteries, as shown in Figure 1, complex components were identified. The traditional three-Gaussian fitting method needs to be generalized to adapt this problem. A modified multi-Gaussian fitting method was proposed to decompose the pulse waveforms from the dogs' femoral arteries. With a confirmation of accurate BP readings from the synchronous recorded invasive intraventricular BP signal, we aimed to accurately determine the relationships between changes in pulse components and varied intraventricular BP values.

## Methods

### Ethics statement

Two healthy beagles (weighing 9–10 kg) were involved in this study for repeated measurements during an anesthetized state. The study obtained full approval from the Animal Care Committee of Chongqing Medical University and all experiments were performed in accordance with the “Guidelines for Ethical Conduct in the Care and Use of Nonhuman Animals in Research” developed by the American Psychological Association. The experiments were conducted at the Affiliated Animal Experiment Center of Chongqing Medical University.

### Data acquisition

**Figure 2:**
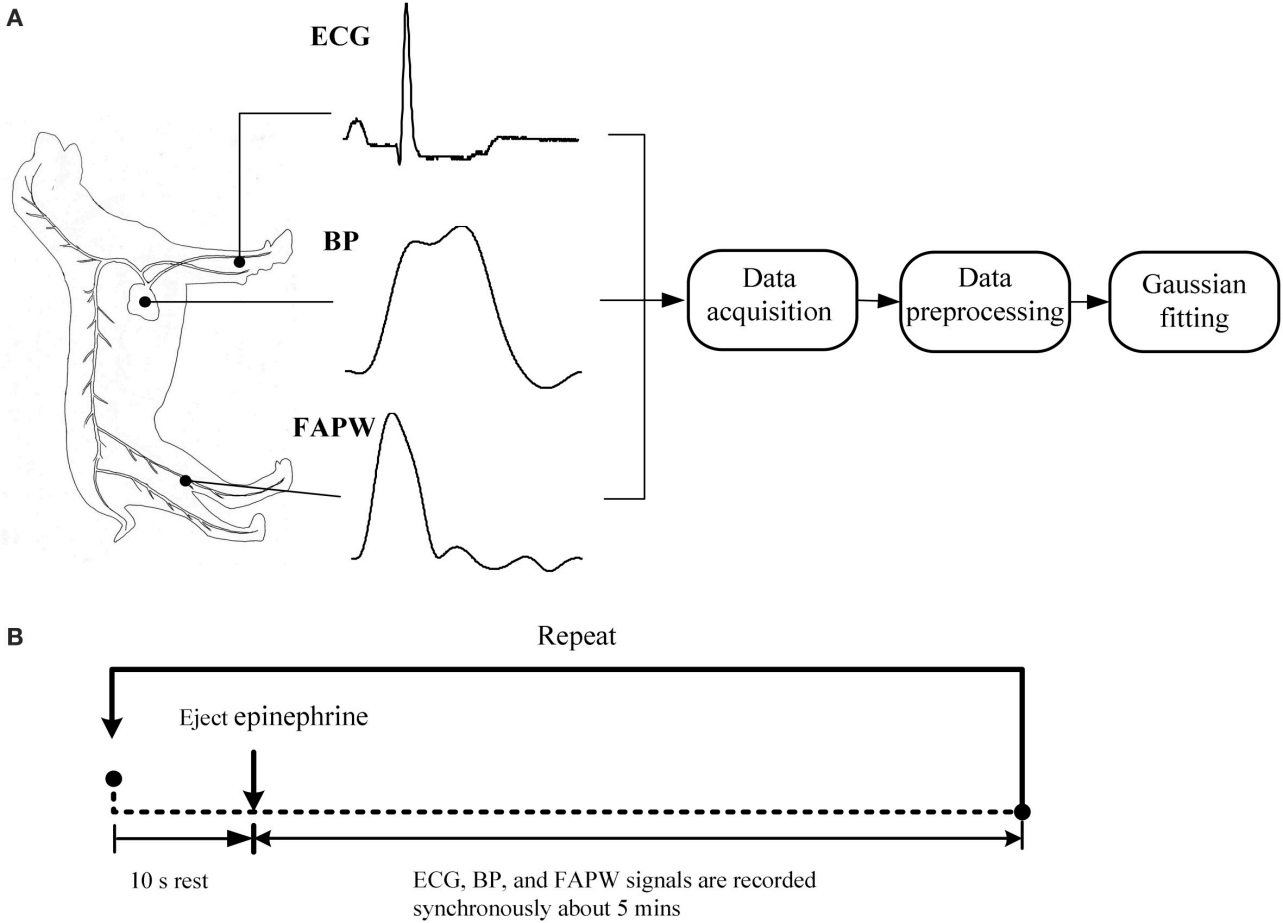
**Schematic diagram and experimental procedure of the measurement system**. **(A)** The standard lead II ECG, blood pressure (BP) in the left ventricle and femoral arterial pulse waveform (FAPW) were synchronously recorded at a sampling rate of 1000 Hz, **(B)** the experimental procedure (This figure was drawn by the author Hong Tang).

The beagles were firstly anesthetized with xylazine (0.2 ml/kg). We then performed the signal recordings while the dogs were in the supine position. A catheter filled with a heparinized solution (500 units/ml) was inserted into the left ventricle via the carotid artery. The catheter was coupled with a high-fidelity BP transducer (MLT0699, ADInstruments, Australia), which was calibrated at standard atmospheric pressure and used to record the invasive intraventricular BP signals. The transducer coupled with fluid filled catheter has a flat frequency response covering animals' heart rate, including rodents (about 10 Hz) according to the technical documents provided by AD Instrument. For producing the varied BP values, epinephrine was ejected, with uncontrolled flow speed, into the beagle's upper limb vein through a path formed by an intravenous infusion of 0.9% saline. This path remained open during the signal recording so that epinephrine could be ejected at any required time. Simultaneously, a photoplethysmogram sensor (MLT1020FC, ADInstruments, Australia) was non-invasively attached to the dogs' femoral arteries to obtain the femoral arterial pulse waveform (FAPW) signals and a standard lead II ECG (ML870, PowerLab 8/30, ADInstruments, Australia) was recorded. All of the three signals were sampled with a sample rate of 1000 Hz, and were converted into digital signals with a 16-bit resolution. The catheter, BP transducer, ECG electrical node, pulse sensor, and all the electrical lines were immobilized during the signal recording to avoid the influence of motion artifacts. The schematic diagram and experimental procedure of the measurement system are shown in Figure 2.

The whole process of signal recording was divided into three stages. In each stage, a different dose of epinephrine was injected and different recording repeats were performed (see Table 1). For each stage and each recording repeat, the signal recording began 10 s prior to the injection of epinephrine and ended until the BP declined to the baseline level (usually 4–6 min). As shown in Table 1, 26 recordings were obtained.

**Table 1:** **The information from signal recording and the number of single cardiac beats in each BP group**.

**Dog**	**State**	**Dose of epinephrine**	**Repeats**	**Record no.**	**Single cardiac beat no**.				**Min. HR**	**Max. HR**	**Aver. HR**
					**Total**	**LBP group**	**MBP group**	**HBP group**			
No. 1	Stage 1	0.5 μg/kg	4	1	412	248	164	0	95	163	128
				2	526	140	319	67	114	160	136
				3	603	216	348	39	105	155	133
				4	623	156	415	52	110	151	133
	Stage 2	1 μg/kg	5	5	775	262	390	123	111	167	136
				6	664	278	284	102	103	164	132
				7	572	336	214	22	100	163	126
				8	558	320	184	54	90	153	123
				9	490	337	121	32	99	141	119
	Stage 3	2 μg/kg	4	10	430	230	129	71	97	176	124
				11	393	201	123	69	98	169	122
				12	411	262	121	28	92	150	118
				13	415	322	83	10	95	147	114
No. 2	Stage 1	0.5 μg/kg	4	14	519	33	300	186	82	173	113
				15	584	16	243	325	103	179	123
				16	608	27	274	307	103	182	125
				17	517	27	186	304	91	177	124
	Stage 2	1 μg/kg	5	18	613	81	159	373	98	187	134
				18	649	174	116	359	79	180	133
				20	615	205	151	259	89	168	128
				21	601	219	155	227	93	167	125
				22	594	228	162	204	95	162	122
	Stage 3	2 μg/kg	4	23	611	160	105	346	89	174	130
				24	598	196	94	308	90	167	128
				25	549	209	91	249	86	156	122
				26	429	211	71	147	88	131	106
	Total	–	–	–	14,359	5094	5002	4263	–	–	–

*LBP group had SBP below 180 mmHg, MBP group had SBP within [180–210] mmHg, and HBP group had SBP above 210 mmHg. Min. HR, minimum heart rate in beats per minute; Max. HR, maximum heart rate in beats per minute; Aver. HR, average heart rate in beats per minute*.

Each recording was first performed by using the pre-processing procedure. The single cardiac beat signals were then extracted from the FAPW signal. These single beat FAPW signals were divided into low, medium, and high BP groups based on the SBP values of the cardiac beats; the low BP group had a SBP below 180 mmHg, the medium BP group had a SBP within [180–210] mmHg and the high BP group had a SBP above 210 mmHg. This grouping permitted us to compare the changes in the pulse waveform between different BP groups.

### Response for epinephrine

**Figure 3:**
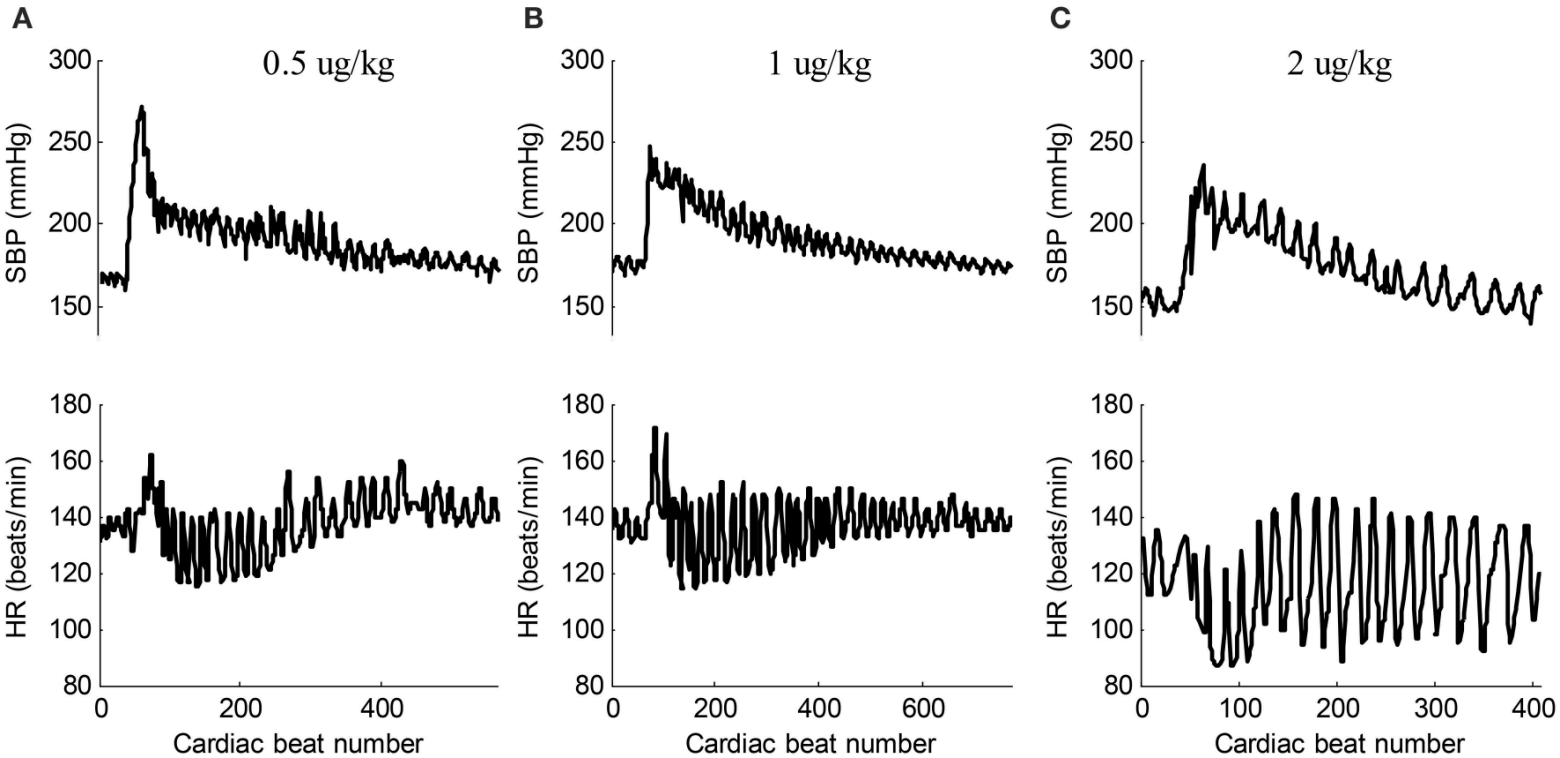
**Typical responses of SBP and HR signals when ejecting different dose of epinephrine. (A)** 0.5 ug/kg (recording No. 2), **(B)** 1 ug/kg (recording No. 5), **(C)** 2 ug/kg (recording No. 12). In each sub-figure, the upper panel shows the SBP signal and the lower panel shows the corresponding HR signal.

Epinephrine, secreted by the medulla of the adrenal glands, is a hormone and a neurotransmitter. It is also produced at the ends of sympathetic nerve fibers and serves as chemical mediators for conveying the nerve impulses to effector organs. Epinephrine increases the heart contractility. Figure 3 shows the typical responses of SBP and heart rate (HR) when ejecting different dose of epinephrine. After ejecting epinephrine, a general observation is that the intraventricular BP increases rapidly. In Figure 3A, the dose of epinephrine was 0.5 μg/kg. The intraventricular BP declined quickly by natural metabolic responses. The doses of epinephrine in Figures 3B,C were 1 μg/kg and 2 μg/kg, respectively. The dog required more time to metabolize this dose of epinephrine. The intraventricular BP declined more slowly compared to the decline following the 0.5 ug/kg dose. In the 26 recordings, a relative large BP range was observed with a minimum SBP of 137 mmHg and a maximum SBP of 271 mmHg, thus inducing a varied BP range of 271 − 137 = 134 mmHg for analyzing the effect of BP on the changes in pulse waveform. To the best of our knowledge, this is the widest BP range used for testing the effect of BP. The local fluctuations in the SBP and HR signals shown in Figure 3 are caused by the dogs' uncontrolled respiration. The large HR changes within the response processes of epinephrine are obvious; it was thus necessary to perform time normalization for each cardiac beat pulse waveform to remove the effect of the HR.

### Data preprocessing

The following data preprocessing steps were applied to the synchronously recorded ECG, BP, and FAPW signals.

**Figure 4:**
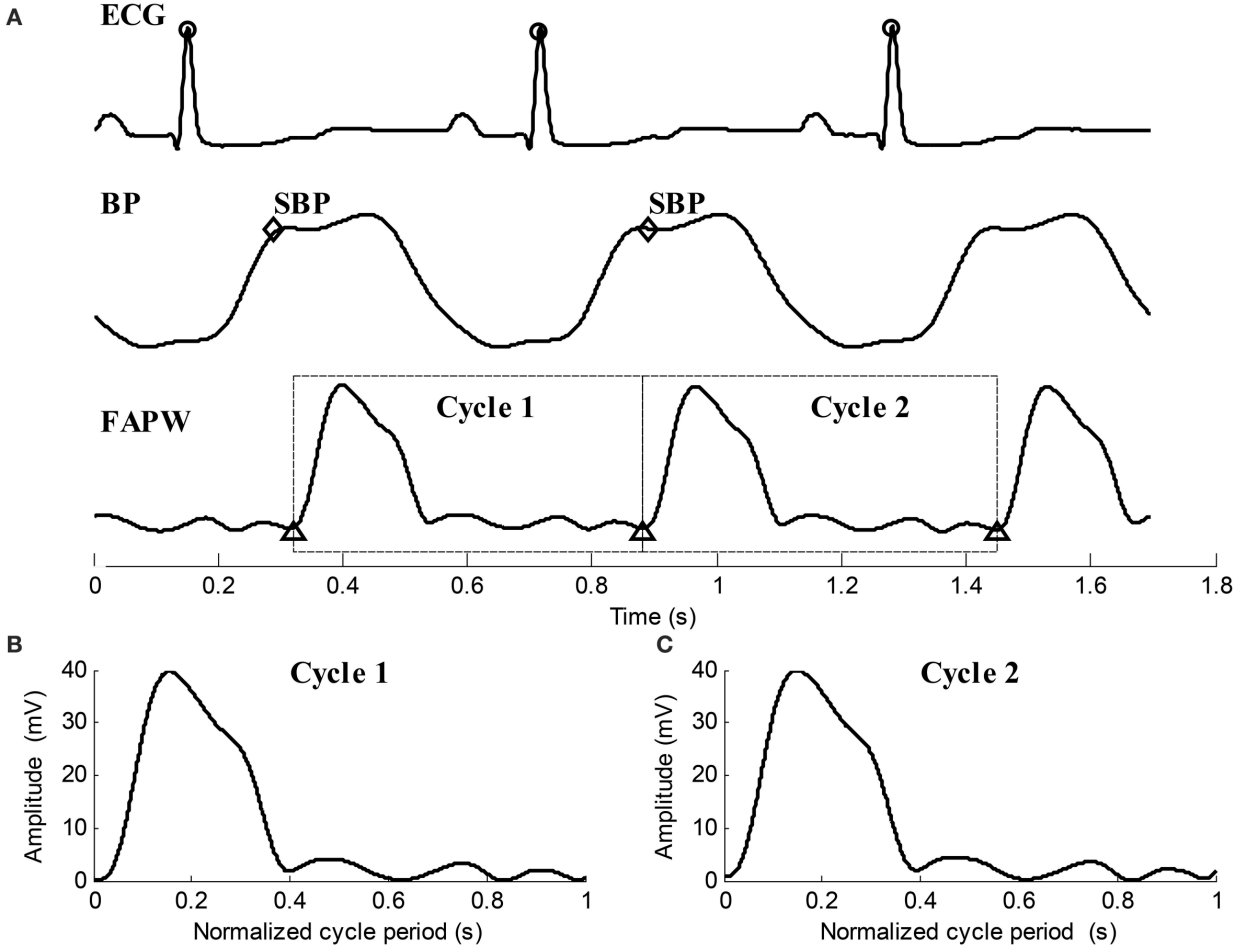
**Illustration examples of data preprocessing for the ECG, BP, and FAPW signals**. **(A)** R-peaks were detected and labeled with “o,” SBP points in each cardiac cycle were indicated by “♢,” Pulse feet of the FAPW signal were denoted as “Δ,” **(B,C)** two continuous FAPW pulse waveforms after cycle period normalization.

1. The slow varying components (0–0.05 Hz) were removed from all three recorded signals.
2. The R-peaks of the ECG were detected using the real-time method proposed in Pan and Tompkins (1985).
3. After locating the R-peaks, the BP signal was segmented into isolated cardiac cycles. The BP value corresponding to the end point of the heart contraction in each cardiac beat was determined to be the SBP value.
4. By locating the R-peaks, the pulse feet of FAPW signals were also detected based on the parametric modeling of the rising edge of pulse waveform (Solà et al., 2009). Each FAPW signal was then separated between the consecutives pulse feet to form single cardiac cycle pulses. Time normalization was performed for each cardiac cycle pulse. Figure 4 provides illustrated examples of the data preprocessing.

### Gaussian fitting

Previous studies (Liu et al., 2013, 2014a,b,c; Wang et al., 2013) have reported that both human carotid and radial pulse waveforms can be accurately and steadily fitted by three-Gaussian curves. However, in the present study, the pulse waveform collected from the dogs' femoral arteries is more complex than those collected from the human carotid and radial arteries; it has up to five components. In addition, for the optimization of results, the previous Gaussian fitting method outputs the Gaussian curves all at once. In this study, we modified the Gaussian fitting method and extracted the Gaussian curves from the pulse waveform one by one until the residual was sufficiently small. The new modified Gaussian fitting method was summarized as follows:

Let *x*(*n*) denote the presently processed pulse waveform. The *k*-th Gaussian curve is defined as:

(1) $$\begin{array}{l} f_{k}\left(a_{k},t_{k},w_{k};n\right)=a_{k}\exp\left(\frac{-{\left(n-t_{k}\right)}^{2}}{w_{k}^{2}}\right) \end{array}$$

Each Gaussian curve was determined by three parameters: the peak height *a*_*k*_, the peak position *t*_*k*_, and the time support width *w*_*k*_. *n* is the normalized time index of the Gaussian curve with *n* = 1, 2, …, 1000. The first separated Gaussian function has the maximum peak height. Therefore, the raw peak position ${\hat{t}}_{k}$ is estimated as the temporal position of the maximum peak.

The objective function for optimization based on Least Square criteria is defined as:

(2) $$\begin{array}{l} J\left(a_{k},t_{k},w_{k}\right)=\overset{1000}{\underset{n=1}{\sum }}{\left(x\left(n\right)-f_{k}\left(a_{k},t_{k},w_{k};n\right)\right)}^{2} \end{array}$$

The optimal *t*_*k*_ must be close to the raw estimated peak position. Thus, the searching range for *t*_*k*_ can be limited to [${\hat{t}}_{k}$ − ξ${\hat{t}}_{k}$ + ξ], where ξ is the small number. In this paper, ξ is set to 5 ms. The parameter *a*_*k*_ is the maximum peak height. However, the searching range for *w*_*k*_ can be set to a reasonable range of [1–150] ms. Many optimization methods can be used here to estimate the optimal parameters. For example, grid searching is the most direct and simplest method, although it is time consuming. Other efficient optimization methods include the Simulated Annealing (Fabian, 1997), the two-stage particle swarm optimizer (Liu et al., 2014a), and the nonlinear least square method or the weighted version (Wang et al., 2013).

The residual after separation of the *k*-th Gaussian curve is:

(3) $$\begin{array}{l} x_{k+1}\left(n\right)=x_{k}\left(n\right)-f_{k}\left(a_{k},t_{k},w_{k};n\right) \end{array}$$

where *x*_1_(*n*) is the original pulse waveform *x*(*n*). If the number of extracted Gaussian curves meets the operator's expectation, the decomposition stops. Otherwise, *k* = *k* + 1, and go to estimate the peak position of new Gaussian function to repeat the procedure.

The root mean square error (*RMSE*) of the final residual is:

(4) $$\;RMSE=\sqrt{\sum_{n\;=\;1}^{1000}x_{k\;+\;1}^{2}(n)/\sum_{n\;=\;1}^{1000}x^{2}(n)}$$

**Figure 5:**
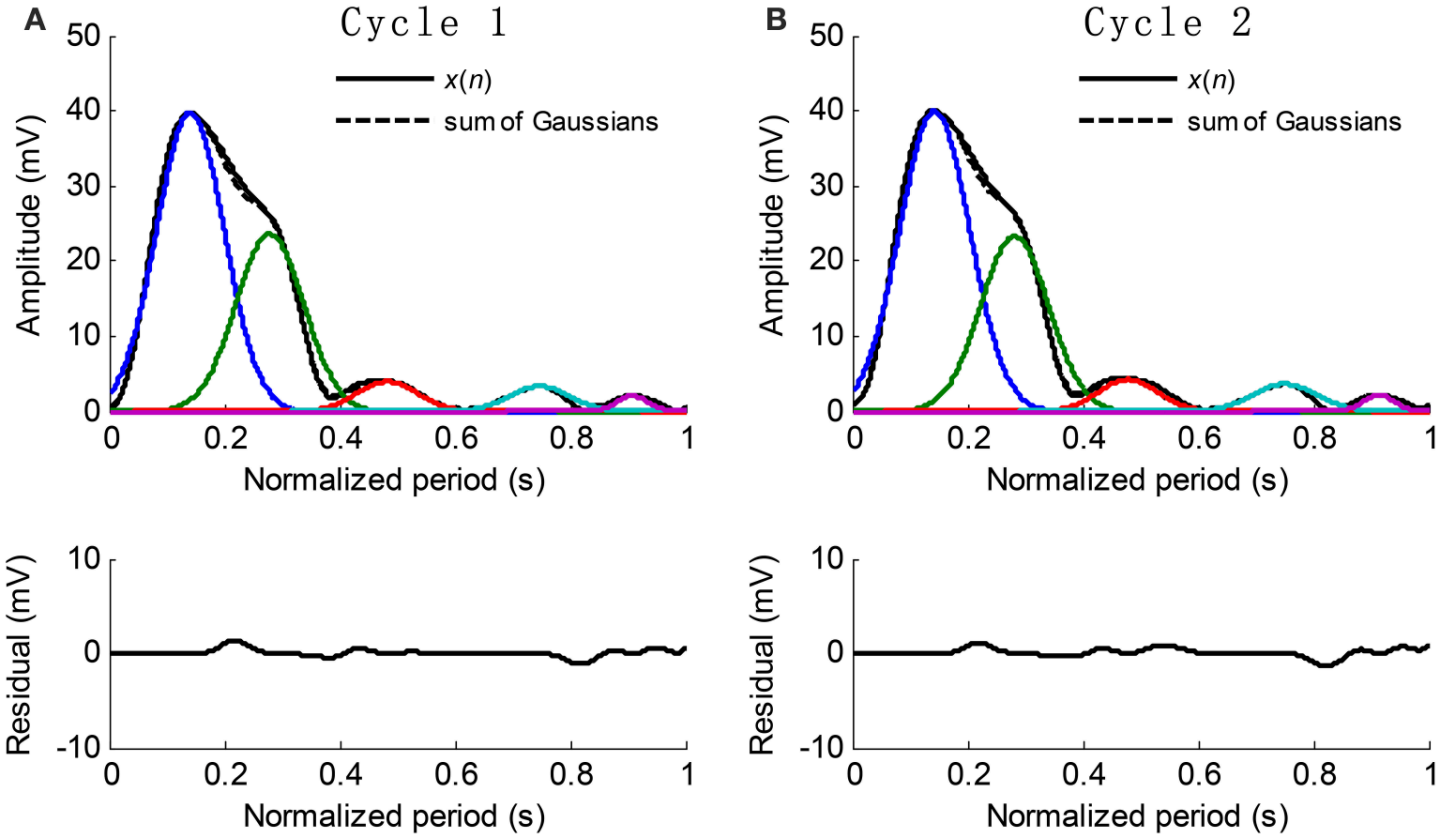
**Examples of Gaussian fitting for the normalized cycle period pulses**. **(A,B)** The extracted Gaussian curves and the corresponding residual.

The extracted Gaussian curves are listed in ascending order according to their peak positions, i.e., *t*_1_ < *t*_2_ < … < *t*_*k*_. Figure 5 shows the examples of waveform fitting. This experiment shows that the *RMSE* is commonly less than 5% when the number of Gaussian curves goes up to five. Therefore, the pulse waveform is characterized by the separated Gaussian curves. Any change in the pulse waveform shape can be theoretically reflected by the varied parameters. It is reasonable to investigate the changes of pulse waveform shape with increased BP based on the obtained parameters.

Previous studies have shown that the first Gaussian curve is usually considered as the forward component and the second Gaussian curve is considered as the main reflected component. In this study, we analyzed the height, position, and time support width of all five Gaussian curves, aiming to disclose the accurate effect of BP on the changes of the components of pulse waveform.

### Data and statistical analysis

First, a linear regression analysis was used to detect the statistical significance of the linear correlation between each Gaussian parameter and the SBP values. Then, the linear regression equations, correlation coefficient *R*-values, *P*-values, and the standard variance of prediction errors (STD) were calculated for each recording. STD was used to measure dispersion of the linear regression.

To further explore the variation of Gaussian parameter within the experimental dogs, we used the method recommended by Bland and Altman (1995a,b). The Gaussian parameter was set as the outcome variable, and the BP values and the experimental subjects as the predictor variables. Experimental subject was treated as a categorical factor using dummy variable. Variance table was used for the regression, which showed how the variability in a Gaussian parameter can be partitioned into components due to different sources. The magnitude of the correlation coefficient (CC) within subjects was the square root of this proportion given in Equation (5). The sign of CC value was given by the sign of the regression coefficient for BP factor.

(5) $$\begin{array}{l} \text{CC within subjects}=\\ \sqrt{\frac{\text{sum of squares for blood pressure}}{\text{sum of squares for blood pressure}+\text{residual sum of squares}}} \end{array}$$

SPSS software package (SPSS Inc.) was employed to perform regression analysis. A value of *p* < 0.05 was considered statistically significant. Finally, the differences between the three SBP groups were tested to determine the effect of BP values on Gaussian parameters.

## Results

### Typical example results from recording No. 9

**Figure 6:**
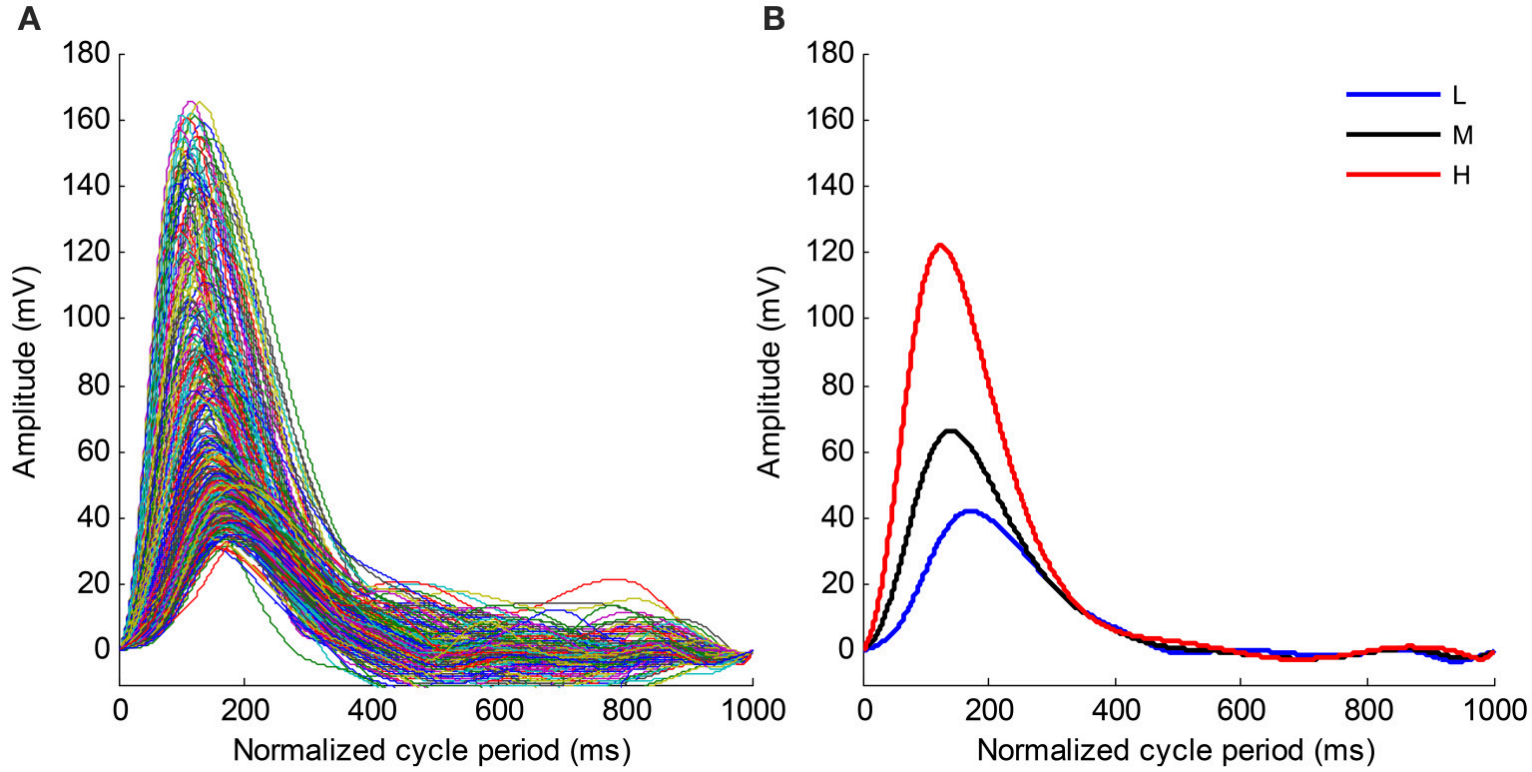
**(A)** Time normalized pulse waveforms for all cardiac beats from the recording No. 9. **(B)** Mean pulse waveforms from three SBP groups. L, Low SBP group; M, Median SBP group; H, High SBP group.

**Figure 7:**
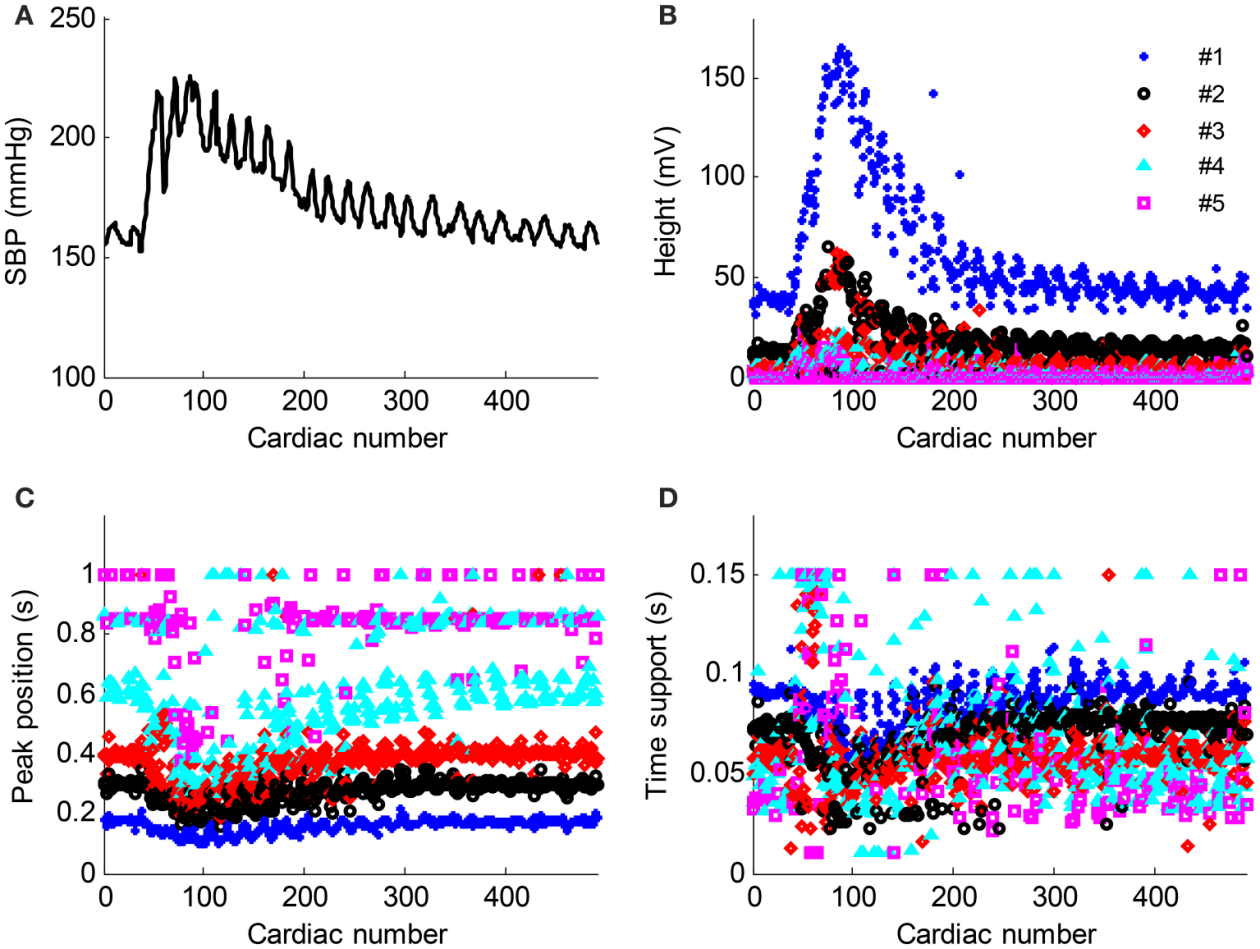
**Parameter evolution of the five Gaussian curves from the recording No. 9 with respect to the cardiac number**. **(A)** Beat-to-beat SBP signal change, **(B–D)** the height, peak position and time support parameters change with respect to the cardiac number. The parameters from the Gaussian #1–#5 are denoted as “_*_,” “o,” “◇,” “△,” and “□,” respectively.

**Figure 8:**
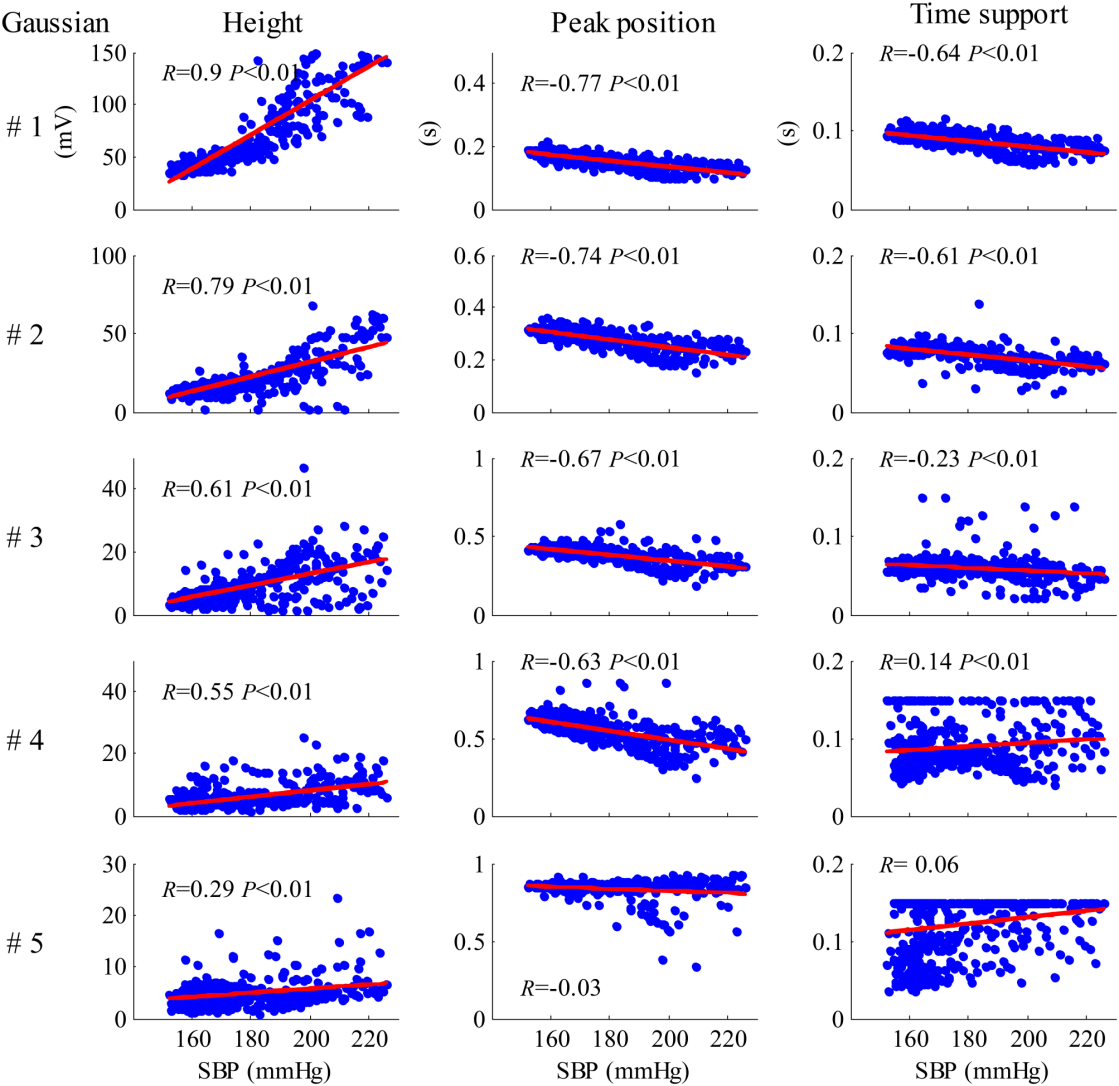
**Results of the correlation analysis between the Gaussian parameter values from each cardiac beat and SBP values for the recording No. 9**.

**Figure 9:**
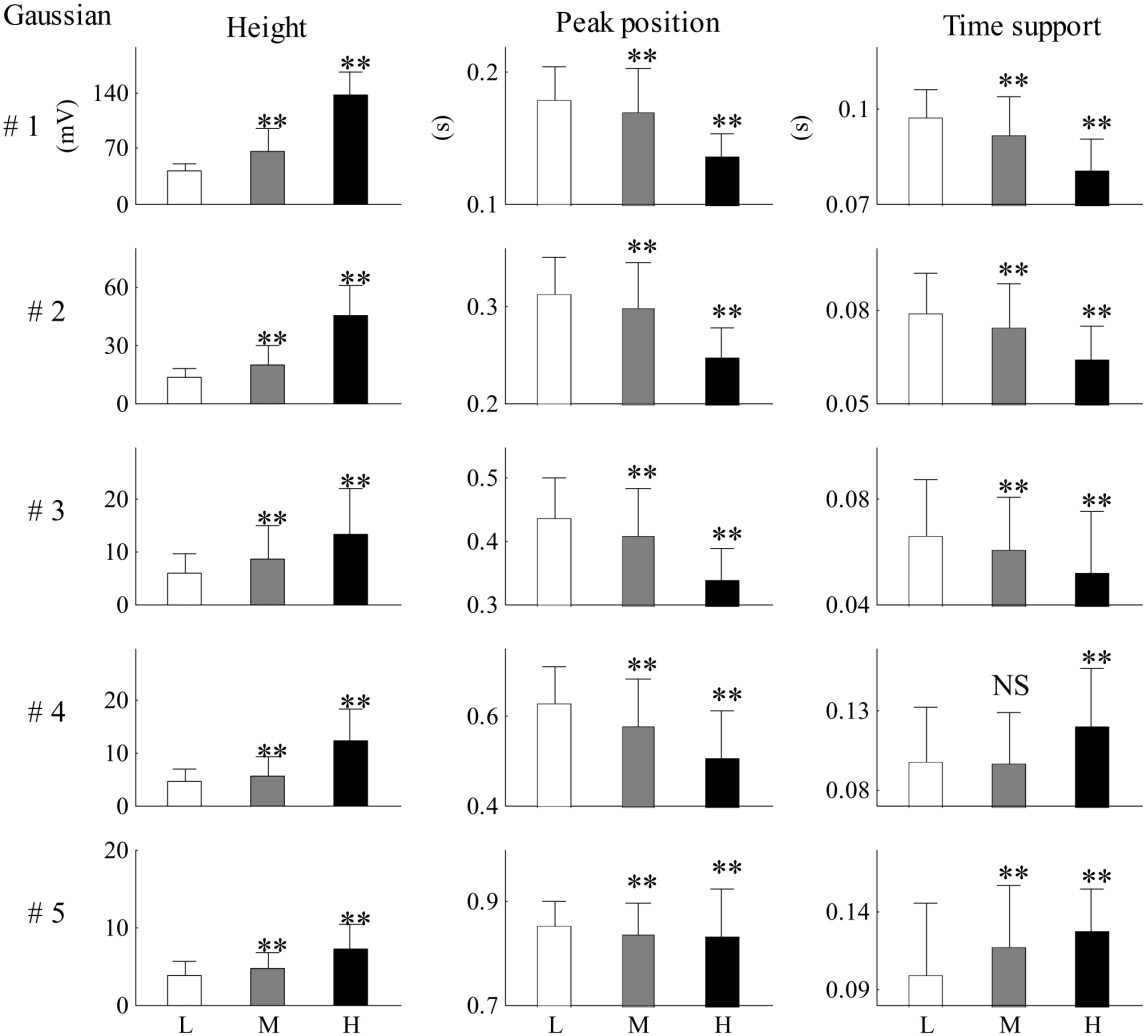
**Mean and SD values for each Gaussian parameter for each SBP group from all 26 recordings**. Compared with the low SBP group, statistical differences were denoted as ^**^p < 0.01, and “NS” (*p* ≥ 0.05). L, low SBP group; M, medium SBP group; H, high SBP group.

**Figure 10:**
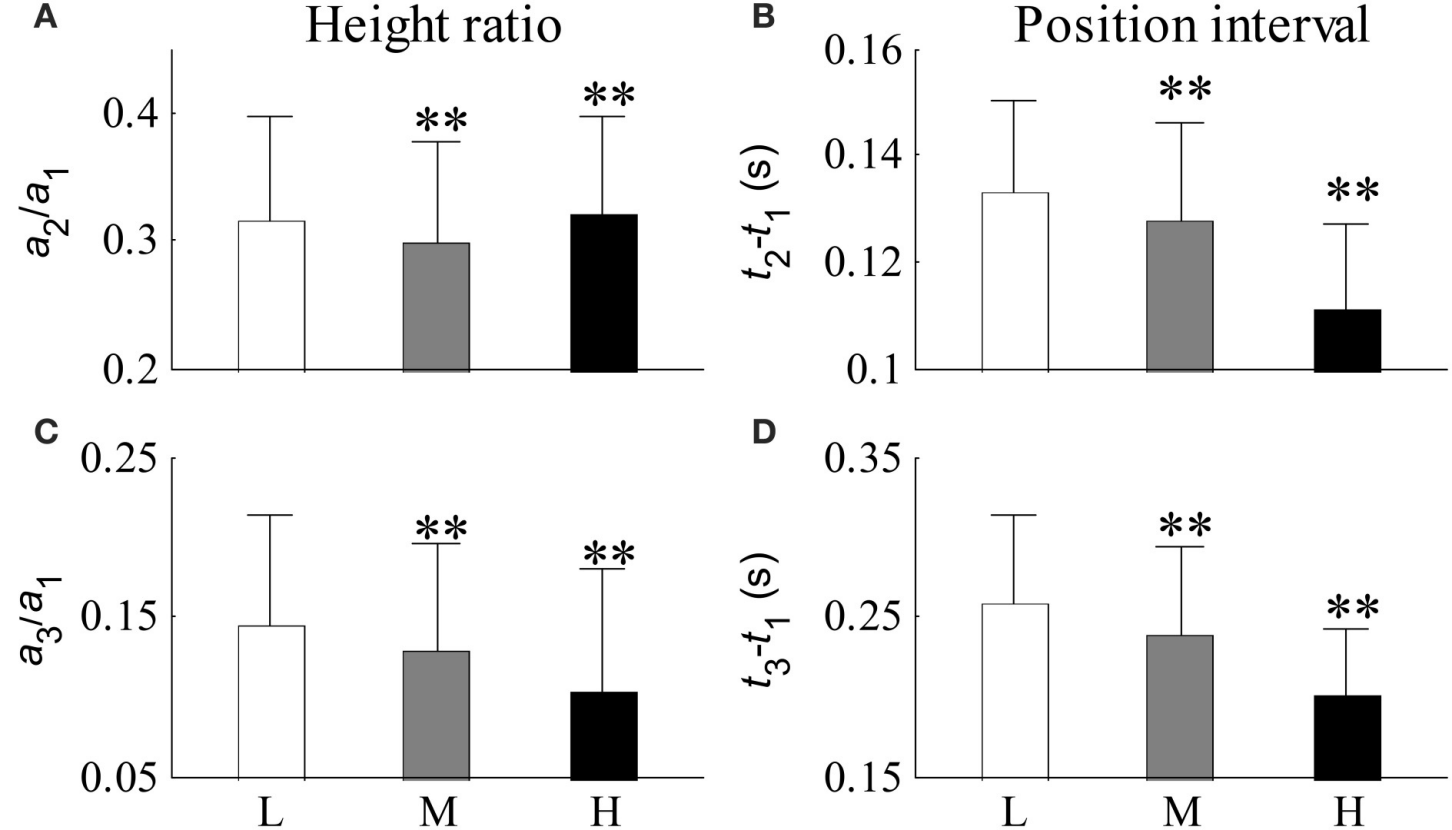
**Relative changes in parameters from the first three Gaussian curves for each BP group**. **(A)** Ratio of *a*_2_ to *a*_1_, **(B)** peak interval between the first two Gaussian curves, **(C)** ratio of *a*_3_ to *a*_1_, and **(D)** peak interval between the first and third Gaussian curves. Compared with the low SBP group, statistical differences were denoted as ^**^p < 0.01, and “NS” (*p* ≥ 0.05). L, low SBP group; M, medium SBP group; H, high SBP group.

Recording No. 9 is selected as a typical recording where the dose of epinephrine was 1 μg/kg. The results analyzed from the typical recording are shown in Figures 6–**8**. Each recording was analyzed following in the same way. The statistical results from twenty-six recordings showing how the Gaussian parameters vary with respect to BP are shown in Tables 2–**4**. The significance of the Gaussian parameters among BP groups is summarized in **Figures 9**, **10**.

**Table 2:** **A summary of the height parameter as a function of SBP**.

**Gaussian**	Height = SBP^*^s + *d*		***R*-values**	**Number of recordings**		**STD (mV)**	**CC within subjects**
	***s***	***d***		***P* < 0.05**	***P* < 0.01**		
No. 1	1.68 ± 0.38	−2.4±78.0	0.85 ± 0.09	26	26	17.1	0.91
No. 2	0.51 ± 0.16	−74.5±31.2	0.74 ± 0.09	26	26	8.5	0.70
No. 3	0.17 ± 0.05	−22.1±6.1	0.51 ± 0.17	26	25	6.0	0.59
No. 4	0.11 ± 0.06	−15.0±10.8	0.50 ± 0.18	26	25	3.8	0.63
No. 5	0.06 ± 0.05	−6.7±6.5	0.40 ± 0.20	26	25	2.5	0.53

*Data are shown in number or mean ± standard deviation*.

Figure 6A shows the time normalized pulse waveforms for all cardiac beats from recording No. 9 and Figure 6B shows the mean pulse waveforms from each SBP group to permit the waveform changes among different BP groups to be observed. When the BP changes from the low SBP group to the high SBP group, the amplitude of the mean pulse waveform increases and the peak position of the mean pulse waveform appear early. Meanwhile, the mean pulse waveform from the high SBP group is sharper than those from the low and medium SBP groups.

The time normalized pulse waveforms from each cardiac beat in Figure 6A was decomposed into five Gaussian curves. Figure 7 shows the corresponding decomposed parameters (height, peak position, and time support) of Gaussian curves as well as the beat-to-beat SBP signal needed to permit the real-time responses of Gaussian parameters to the BP changes observed. When SBP increased, the height parameters increased while the peak position and time support parameters decreased.

Figure 8 shows the results of the correlation analysis between the Gaussian parameter values from each cardiac beat and the SBP values for recording No. 9. As shown in Figure 8, all five height parameters have strong positive correlations with SBP (all *p* < 0.01). Meanwhile, all five peak position parameters have negative correlations with SBP, but the correlations from only the first four parameters are statistically significant (*p* < 0.01). For the time support parameters, the correlations from the first three parameters were negative and the correlations from the last two parameters were positive.

### Results from all 26 recordings

Tables 2–**4** summarize the results of the regression analysis between each type of Gaussian parameter (height, peak position, and time support) and the SBP values. Regression equations for each Gaussian parameter were obtained from the recordings were statistically significant (*p* < 0.05). The numbers of recordings with statistical significance were also given.

Regression analysis for height parameter, as summarized in Table 2, showed that the height parameters from the five Gaussian curves were significantly related to SBP (all *p* < 0.05) for all 26 recordings, with decreasingly significant correlation coefficient *R*-values from the first to the fifth Gaussian curves. The regression equation for the first Gaussian height parameter had the maximum slope and correlation coefficient. Absolute regression slopes also decreased from the first to the fifth Gaussian curves. From the first to the fifth Gaussian curves, the parameter dispersion from the linear regression equations became smaller, indicated by the decreased STD values.

Table 3 summarizes the regression analysis results between the peak position parameters and the SBP values. The peak position parameters from the first four Gaussian curves were highly negatively correlated (|*R|* > 0.5) with increased SBP, with the mean absolute slope increased from 11.0 to 27.0. The parameter dispersion from the linear regression equations became larger from the first to the fourth Gaussian curves, indicated by the increased STD values.

**Table 3:** **A summary of the peak position parameter as a function of SBP**.

**Gaussian**	Peak position = SBP^*^10^−4*^s + *d*		***R*-values**	**Number of recordings**		**STD (s)**	**CC within subjects**
	***s***	***d***		***P* < 0.05**	***P* < 0.01**		
No. 1	−11.0±3.5	0.32 ± 0.09	−0.65±0.17	25	25	0.03	−0.72
No. 2	−15.0±4.1	0.55 ± 0.10	−0.65±0.15	25	25	0.04	−0.58
No. 3	−21.8±8.5	0.82 ± 0.23	−0.54±0.16	26	26	0.07	−0.47
No. 4	−27.0±8.0	1.20 ± 0.18	−0.50±0.16	26	26	0.09	−0.38
No. 5	−8.2±8.0	1.20 ± 0.19	−0.21±0.15	24	24	0.07	−0.10

Table 4 summarizes the regression analysis results between the time support parameters and SBP values. The time support parameters from the first three Gaussian curves were negatively correlated with SBP, while those from the last two Gaussian curves were positively correlated with SBP. However, only 14 recordings were significantly related to SBP values for the fourth time support parameter. From the first to the fifth Gaussian curves, the parameter dispersion from the linear regression equations increased.

**Table 4:** **A summary of the time support parameter as a function of SBP**.

**Gaussian**	Time support = SBP^*^10^−4*^s + *d*		***R*-values**	**Number of recordings**		**STD (s)**	**CC within subjects**
	***s***	***d***		***P* < 0.05**	***P* < 0.01**		
No. 1	−4.1±1.1	0.17 ± 0.03	−0.60±0.12	26	26	0.02	−0.69
No. 2	−3.4±0.5	0.14 ± 0.02	−0.42±0.12	25	25	0.02	−0.36
No. 3	−3.3±4.1	0.13 ± 0.09	−0.20±0.11	24	22	0.03	−0.23
No. 4	3.1 ± 1.5	0.06 ± 0.04	0.14 ± 0.06	15	14	0.04	0.15
No. 5	7.2 ± 4.6	−0.02±0.10	0.25 ± 0.15	26	25	0.05	0.15

In addition, the CC values within experimental dogs were also reported in Tables 2–4. For each type of Gaussian parameters (i.e., height, peak position and time support), the CC values (absolute values) steadily decreased from the first to the fifth Gaussians, indicating the increase of subject variability if more and more Gaussian functions were involved in the analysis. However, it is worth to note the all CC values from the first Gaussian were at high levels (0.91, −0.72, and −0.69 respectively), suggesting the high repeatability between subjects.

### Comparison between the three SBP groups

Figure 9 shows the mean and SD values for each Gaussian parameter for each SBP group from all 26 recordings. Compared with the low SBP group, both the medium and the high SBP groups had significantly larger height values and had significantly smaller peak position values. Furthermore, the time support parameters in both the medium and the high SBP groups significantly decreased in the first three Gaussian curves but significantly increased in the last two Gaussian curves. All significances had the *P*-values of *p* < 0.01, except the difference in the time support parameter between the low and medium SBP groups from the fourth Gaussian curve, which had a *p* ≥ 0.05. In general, for the first three Gaussian curves, with increasing BP, increased height, and decreased position and time support.

Figure 10 shows the relative changes in parameter results from the first three Gaussian curves for each SBP group. Figures 10A,C shows the height ratios between the second and first Gaussian curves (*a*_2_/*a*_1_) and between the third and first Gaussian curves (*a*_3_/*a*_1_) respectively. Figures 10B,D shows the peak intervals between the second and first Gaussian curves (*t*_2_ −*t*_1_) and between the third and first Gaussian curves (*t*_3_ − *t*_1_) respectively. Compared with the low SBP group, significant decreases from the indices of *a*_2_/*a*_1_, *a*_3_/*a*_1_, *t*_2_ − **t**_1_, and *t*_3_ − *t*_1_ were observed in the median SBP group; the decreases from the indices of *a*_3_/*a*_1_, *t*_2_ − *t*_1_, and *t*_3_ − *t*_1_ were even more significant in the high SBP group (all *p* < 0.01). However, index *a*_2_/*a*_1_ showed a significant increase in the high SBP group (*p* < 0.01) compared to the results from the low SBP group.

### Results of without normalizing cycle period

It was observed in Table 1 that the heart rate varied much in a recording. The changes of Gaussian parameters presented previously in this article were analyzed with normalizing the cycle period to reduce heart rate effect. It is also interesting to investigate the changes without normalizing the cycle period. The linear time scaling operation has no effect on the height parameter. So, it is reasonable to conclude that the changes of height parameter without normalizing cycle period are same to those with normalizing cycle period. So, they are omitted here. Tables 5, 6 summarized the changes of position and time support parameter. Comparison between Tables 3, 5 showed that the change rates of the peak position with normalizing cycle period were greater than those without normalizing. However, their correlation coefficients were of little difference. Observation to Tables 4, 6 also showed that the change rates of time support of with normalizing had the similar conclusion. This is easy to understand because the linear time scaling extends the cycle period. So, the change rates of time domain parameters, peak position and time support, become greater.

**Table 5:** **A summary of the peak position parameter as a function of SBP without normalizing cycle period**.

**Gaussian**	Peak position = SBP^*^10^−4*^s + *d*		***R*-values**	**Number of recordings**		**STD (s)**
	***s***	***d***		***P* < 0.05**	***P* < 0.01**	
No. 1	−2.46±2.24	0.12 ± 0.04	−0.66±0.22	26	26	0.01
No. 2	−5.18±2.74	0.24 ± 0.05	−0.64±0.21	26	26	0.02
No. 3	−9.50±3.85	0.38 ± 0.09	−0.53±0.14	26	26	0.04
No. 4	−13.81±1.51	0.58 ± 0.11	−0.47±0.15	25	25	0.07
No. 5	−13.56±9.83	0.69 ± 0.20	−0.54±0.36	17	15	0.06

*Data are shown in number or mean ± standard deviation*.

**Table 6:** **A summary of the time support parameter as a function of SBP without normalizing cycle period**.

**Gaussian**	Time support = SBP^*^10^−4*^s + *d*		***R*-values**	**Number of recordings**		**STD (s)**
	***s***	***d***		***P* < 0.05**	***P* < 0.01**	
No. 1	−1.21 ± 0.39	0.07 ± 0.01	−0.72±0.13	26	26	0.01
No. 2	−1.81 ± 1.32	0.07 ± 0.03	−0.46±0.23	26	26	0.01
No. 3	−0.93 ± 4.5	0.06 ± 0.09	−0.19±0.09	18	17	0.02
No. 4	−2.47 ± 3.57	0.11 ± 0.07	−0.14±0.06	17	12	0.04
No. 5	2.07 ± 4.94	0.20 ± 0.10	0.21 ± 0.12	21	18	0.04

*Data are shown in number or mean ± standard deviation*.

## Discussions and conclusions

With the help of the synchronously recorded invasive BP signal from the left ventricle, this study extracted 15 parameters from five modeled Gaussian curves for the dogs' FAPW signals, and thus providing the opportunities to disclose the accurate effect of BP on the changes in the morphology of pulse waveform. More importantly, a relatively large BP range was induced by ejecting different doses of epinephrine into the experimental beagles, giving a firm foundation for reproducibility. The main finding of the current study is that with increasing SBP, the amplitudes of the first two components increased, while the peak position and time support parameters decreased. By increasing SBP, the amplitude ratio between the first two components decreased, and the peak intervals between the first two components also decreased. The amplitude ratio between the first two components changed irregularly.

Changes in pulse waveform shape are traditionally accepted as an effective indictor of cardiovascular system health. Previous studies have shown that the pulse waveform reflection changed with hypertension (O'Rourke and Nichols, 2005; Westerhof et al., 2006; Laurent and Boutouyrie, 2007; Hermeling et al., 2010; Baruch et al., 2011; Gu et al., 2014; Liu et al., 2014a). However, the BP data from previous studies was seldom collected invasively. In addition, varied BP values were usually from different subjects, which inevitably included the individual factor on the measurement results. In the present study, the invasive intraventricular BP signal was recorded by directly inserting a catheter into the dogs' left ventricles, to obtain accurate real time BP values. These BP values provide good foundation for quantitatively evaluating changes in pulse waveform shape under different BP levels.

The first Gaussian curve is commonly considered as the forward component of arterial pulse due to the ejecting blood function of the left ventricle, while the other Gaussian curves are likely linked with the different reflection sites within the arterial system. In this study, the first Gaussian curve (#1) had a relatively higher peak amplitude, narrower waveform shape, and occurred earlier with an increase in BP. The physiological explanation for this phenomenon is that the contractility of heart becomes stronger after injecting the epinephrine and thus the blood is pumped into the artery with a stronger force. Consequently, the pulse had a steep upstroke and the peak position of the forward component occurred early.

The peak positions of the second to the fifth Gaussian components occurred significantly earlier with an increase in BP (all *p* < 0.01). The absolute slopes of the regression equations between the peak position parameters and SBP values markedly increased from the first to the fourth Gaussian curves, indicating that the time intervals between the components become shorter with an increase in BP. This result is consisted with the previous conclusion (Baruch et al., 2011) when the time interval between the first two Gaussian components was also observed to be shorter with an increased BP. The early occurrence of the Gaussian waves may be attributed to the decrease in arterial elasticity with BP increases.

In conclusion, the current study demonstrates that higher BP levels cause higher pulse amplitude and earlier appearances of Gaussian components in comparison to normal BP level. It demonstrates that the changes of Gaussian parameters can reflect the changes in femoral pulse waveform caused by the increased blood pressure. These findings are clinically important, and may have further contribution in understanding the underlying changes of arterial pulse properties at different BP levels, evaluating pulse reflections (if the Gaussian components are proved to correspond reflections in the future), accessing the well-being of cardiovascular system.

Some potential limitations should be addressed. First, the use of only 2 dogs is a large but evitable limitation. In this study, we aimed to explore the relationship between the Gaussian waveform parameters and the BP levels. Thus, the stable BP levels from the experimental dog are essential. We achieved this point by acquiring repeatable measurements on the same experimental dog at the same BP levels, as well as repeating the measurements at different BP levels, under the hypothesis that the same BP level from the same experimental subject did not significantly change the PPG waveforms, i.e., did not significantly change the obtained Gaussian waveform parameters. During the measurements, we started a new measurement until the experimental dog's BP level came down to the baseline to ensure the measurements meet independence as much as possible. Meanwhile, CC absolute values (see Tables 2–4) from the first Gaussian were all higher than 0.69, suggesting the high repeatability between subjects. However, the generalization on this study's conclusion is open to question, and further study in large samples would be useful. Secondly, we used the BP values directly measured from the left ventricle as the reference BP levels. It is worth to note that this method only gives valid measures of systolic BP since the left ventricle is effectively uncoupled from the arterial circulation during diastole due to valve closure. We did not obtain the actual diastolic BP values. Thus, this study only reflects the changes of Gaussian components with the increased systolic BP. The changes with diastolic BP are still unknown.

## Author contributions

HT and CL designed the study. HT collected the data. HT, CL performed the data and statistical analyses, interpreted the results, and drafted and reviewed the manuscript. HT and CL obtained the funding for this study.

### Conflict of interest statement

The authors declare that the research was conducted in the absence of any commercial or financial relationships that could be construed as a potential conflict of interest.
